# Long Term Rigid Retained Foreign Object After Breast Augmentation: A Case Report and Literature Review

**DOI:** 10.3389/fsurg.2021.725273

**Published:** 2021-10-12

**Authors:** Coral Franco, Anner Moskovitz, Iuliana Weinstein, Samuel Kwartin, Yoram Wolf

**Affiliations:** ^1^Plastic Surgery Unit, Hillel Yaffe Medical Center, Rappaport Faculty of Medicine, Technion, Haifa, Israel; ^2^The Ruth and Bruce Rappaport Faculty of Medicine, Technion Israel Institute of Technology, Haifa, Israel; ^3^Department of Radiology, Tel Aviv Sourasky Medical Center, Sackler School of Medicine, Tel Aviv University, Tel Aviv, Israel; ^4^Dr. Wolf Clinic, Private Practice, Tel Aviv, Israel

**Keywords:** retained foreign object, patient safely, breast augmentation, syringe coverage, case report

## Abstract

**Introduction:** Retained foreign object (RFO) is a rare iatrogenic complication. This article presents an unprecedented case of a plastic RFO post-augmentation mammoplasty.

**Case Presentation:** We present the case of a 32-year-old woman, 8 years after breast augmentation surgery, with a 4 year history of a palpable migrating mass in the superior lateral quadrant of her right breast with fluctuating levels of pain. Imaging studies included mammography tests, sonographic examinations, a Magnetic Resonance Imaging scan, and a Computed Tomography scan, all of which did not identify any pathological findings. Exploratory surgery discovered a syringe-tip cover in the implant pocket.

**Conclusion:** Persistent complaints and symptoms accompanied by non-specific imaging studies warrant escalation of diagnostic methods, in line with a high awareness for the possibility of an RFO. As pocket lavage is a common practice in various surgeries, this report can serve as a valuable reminder for surgical teams to account for syringe covers and other disposable items at the end of all operations.

## Introduction

Several cases of retained foreign objects (RFO), namely gauzes and pads, were described in the literature after breast augmentation operations (1–5). Yet, to the best of our knowledge, a residual rigid plastic RFO discovered years after a breast augmentation has never been reported yet. Retained objects may either be asymptomatic or present with clinical manifestations shortly or long after surgery. Diagnosis depends on post-surgical imaging, the inflammatory response elicited, and the mass sensation created by the object (6). In the case presented here, the isolated mass palpation with no inflammatory response made a challenging diagnosis.

## Case Presentation

A 32-year-old female patient had undergone a bilateral breast augmentation operation 8 years prior. Her main complaint was a sensation of a palpable mass in the superior lateral quadrant of her right breast. The mass fluctuated with time, disappeared and re-appeared in other quadrants of the breast. During the last 4 years the mass was accompanied by a sensation of mild pain that at times exacerbated and subsided. No disturbances in the shape or size of the breasts were noted.

Past medical history revealed no significant health conditions or previous surgery, with no known allergies or medication usage.

The primary breast augmentation was performed in 2012 by a board-certified Plastic Surgeon. The patient had undergone a subpectoral bilateral breast augmentation with round silicone gel implants.

Physical examination revealed a rigid, mobile, and difficult to palpate mass, sized ~1.5 cm, positioned under the upper lateral edge of the Pectoralis Major muscle, with an unclear sensation of crackling. A sharp localized pain followed the palpation of the mass. Physical examination revealed no additional findings such as axillary lymph nodes and breasts masses.

**Figure 1:**
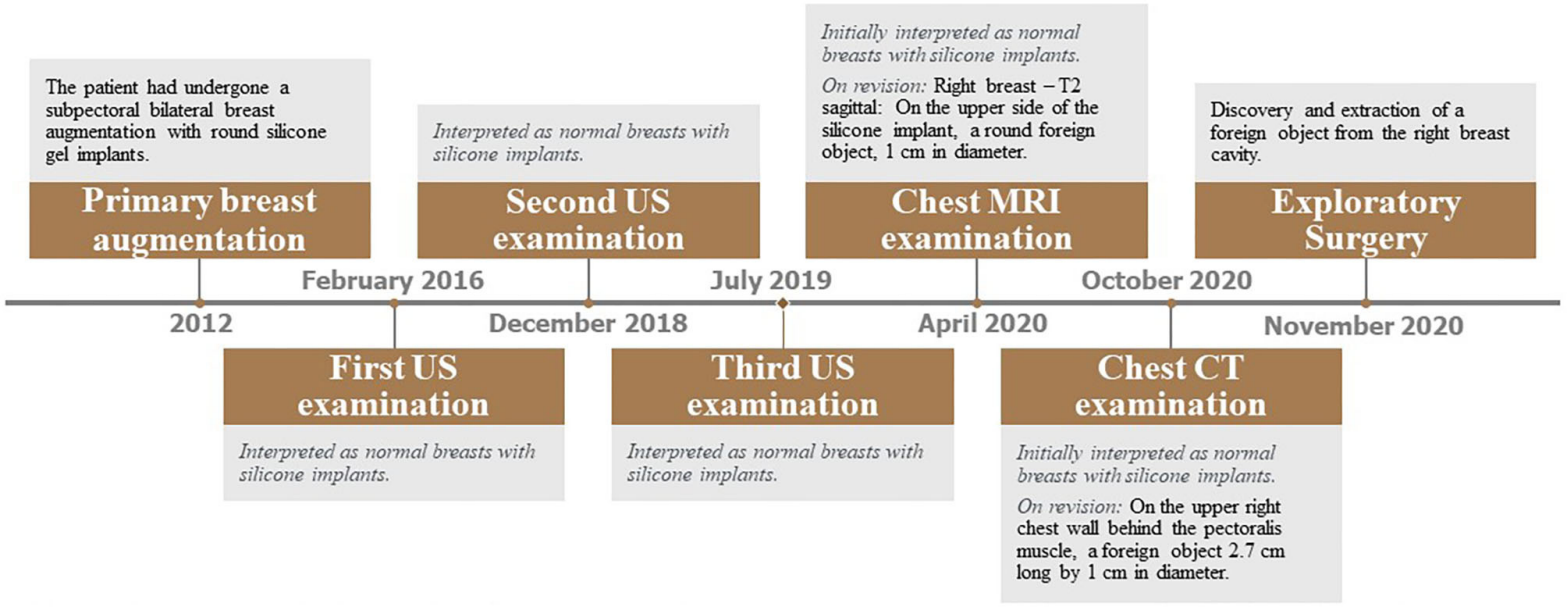
Timeline of relevant data from the episode of care. CT, Computed Tomography; MRI, Magnetic Resonance Imaging; US, Ultrasound.

**Figure 2:**
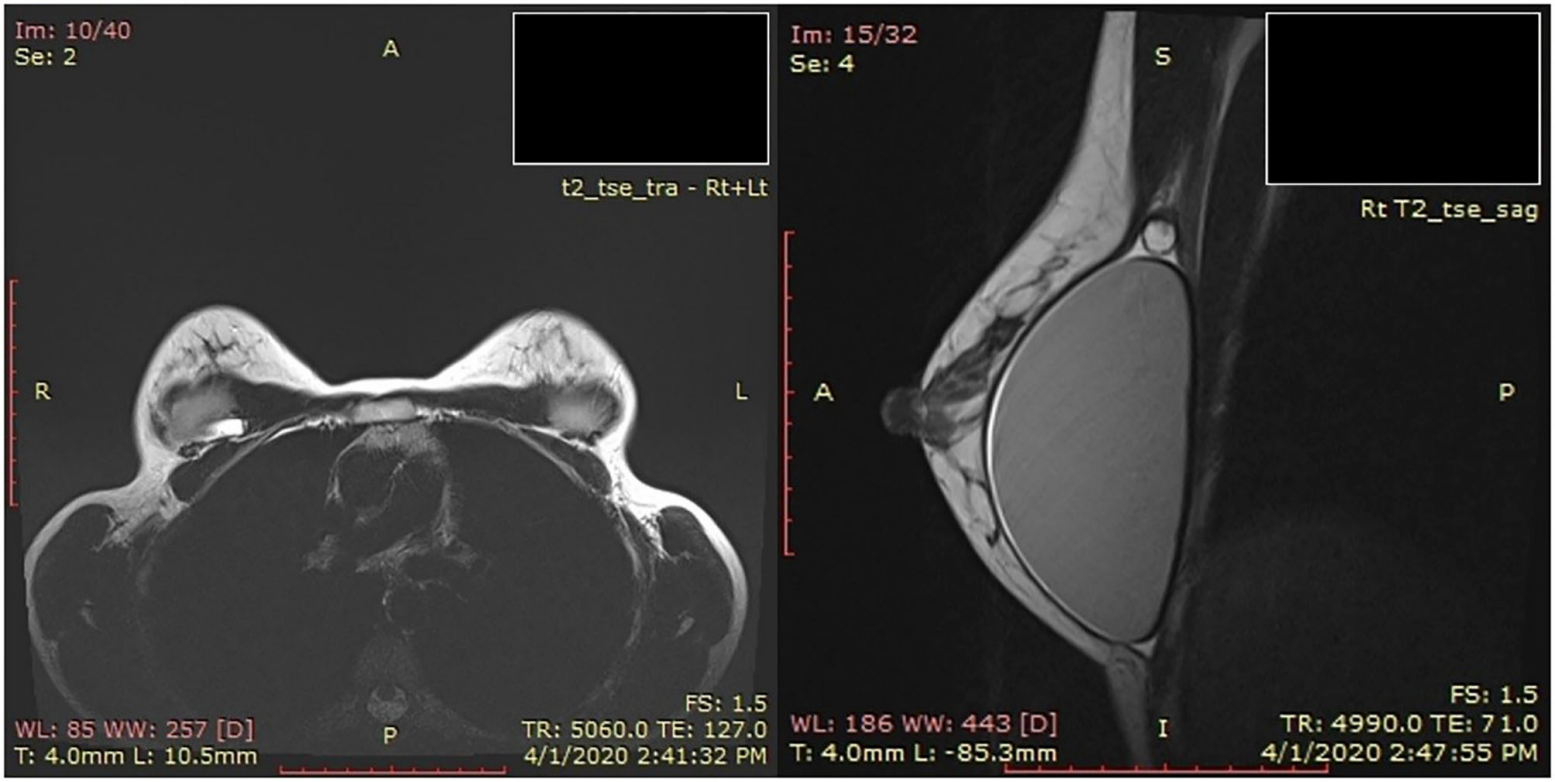
Magnetic Resonance Imaging (MRI) of the breasts. Right breast – T2 sagittal: On the upper side of the silicone implant, a round foreign object, 1 cm in diameter.

**Figure 3:**
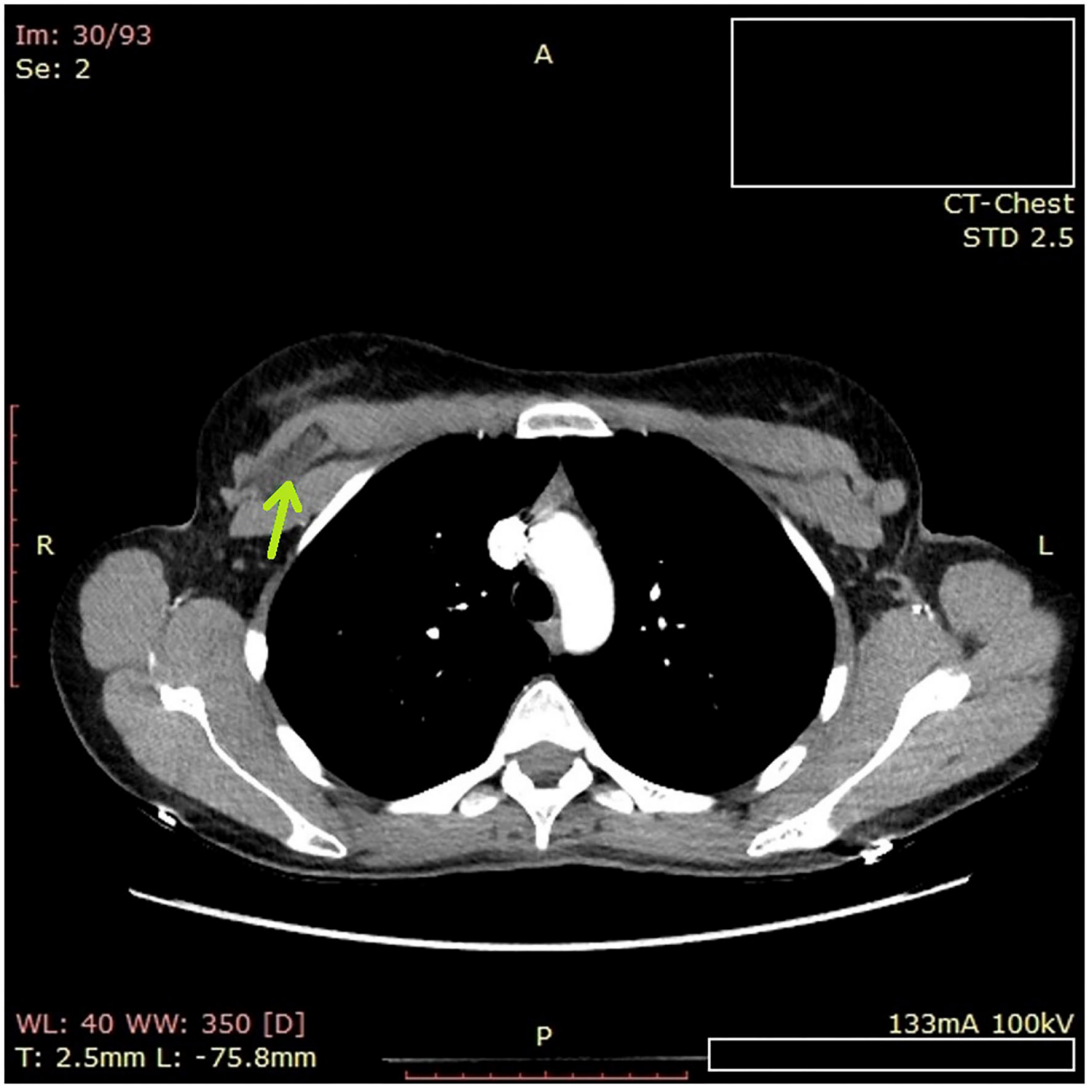
Computed tomography (CT) of the chest. On the upper right chest wall behind the pectoralis muscle, a foreign object 2.7 cm long by 1 cm in diameter.

A timeline describing the radiographic tests performed is shown in Figure 1. Imaging examinations performed over 4 years (2016–2020) included two mammography tests, four sonographic (US) examinations, one Magnetic Resonance Imaging (MRI) scan (Figure 2) including silicone series, and one Computed tomography (CT) scan (Figure 3). Interpretations of the tests results described variable benign findings, with no specific pathology or suspicion for a foreign object.

A re-interpretation of the MRI examination by a board-certified radiologist, who specializes in breast imaging, described a small “non-silicon” object in the right breast suspected to be a localization of fluid.

The patient's complaints and the findings on physical examination were not compatible with a fluid localization, and therefore percutaneous aspiration was not considered appropriate.

**Figure 4:**
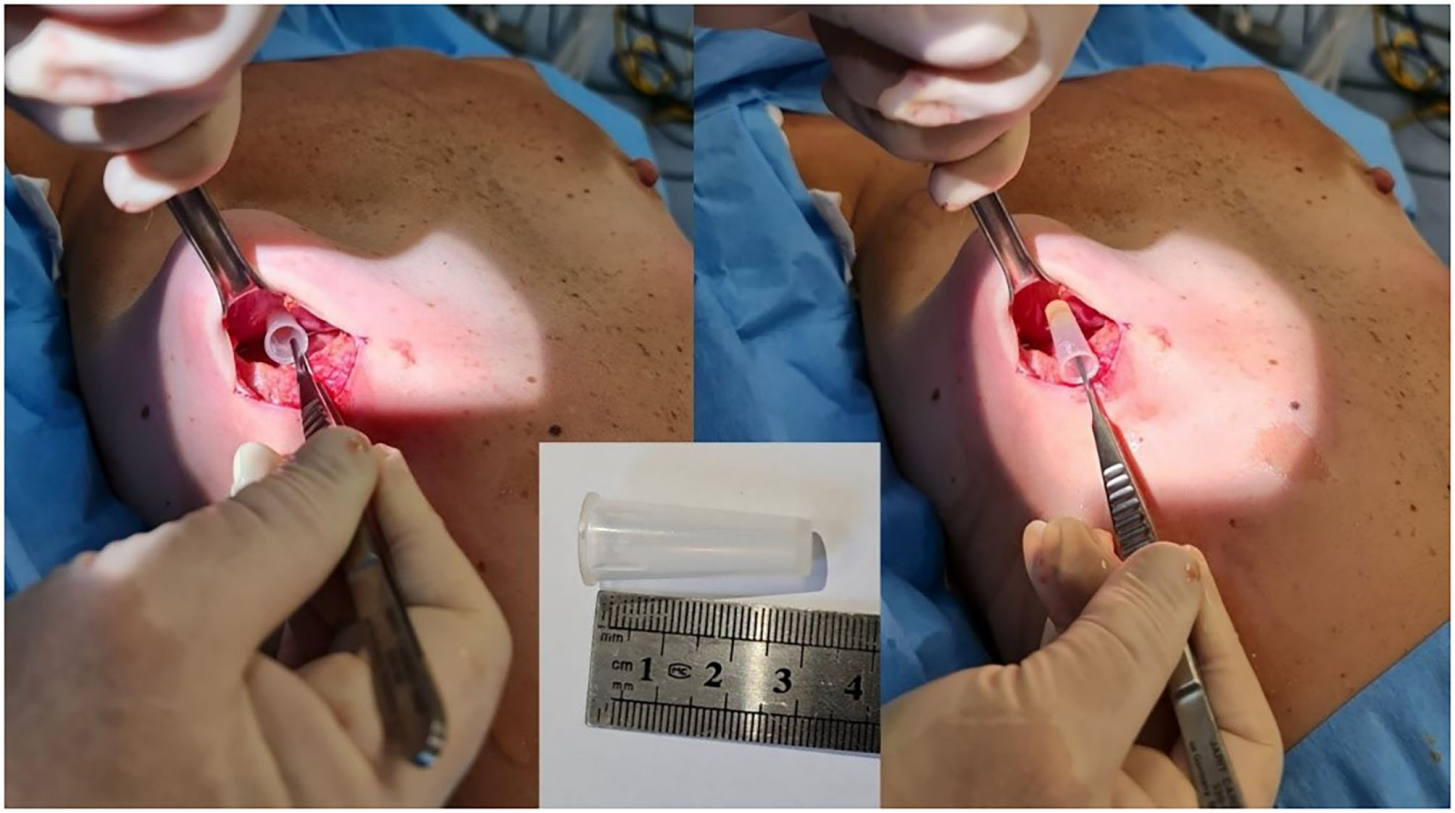
Intra-operative picture. Extraction of a foreign object from the right breast pocket.

Subsequently, exploratory surgery was performed. This operation combined a bilateral examination of the breast implant pockets followed by implant replacement. Implant pockets examination revealed a foreign object in the implant pocket of the right breast. The object was identified as an intact plastic cap of a 60 mL Janet syringe, commonly used for breast augmentation pocket lavage (Figure 4). The capsule was soft with no signs of infection or inflammation, and breast implants were inserted bilaterally. The post-operative period was uneventful, and the patient was discharged on the following day.

The radiologist who performed the re-interpretation of the MRI scan retrospectively reviewed the prior examinations. Her findings were:

- US examinations from February 2016 and July 2019 described normal breasts with silicone implants.
- MRI examination from April 2020 (Figure 2) – Right breast – T2 sagittal: On the upper side of the silicone implant, a round object, 1 cm in diameter, was found.
- Chest CT examination from October 2020 (Figure 3) – A foreign object located on the upper right chest wall behind the pectoralis muscle. The size of the item was 2.7 cm long by 1 cm in diameter.

## Discussion

Retained surgical objects are rare iatrogenic complications in surgery, with an overall estimated occurrence rate of 1.32 in 10,000 procedures (7). The several reported cases of RFO comprise mainly of gossypibomas, retained surgical gauze, following breast augmentation surgery (1–5). A literature search did not reveal any other types of solid foreign bodies discovered following breast augmentation.

Duration of time to diagnosis and clinical manifestations are closely related and divide RFO cases into three clusters. In about one-third of patients, detection is immediate, due to acute symptoms such as pain or findings on routine post-operative imaging. The second cluster, comprising nearly half of the patients, occurs after the initial 24 h and within 3 months post-operatively and presents with symptoms related to infection of the retained object. The third group, the remainder of patients, present after a long delay with a mostly asymptomatic course, either by an incidental finding on imaging studies or as an unexplained mass (6). The case reported here belongs to the third group, with an asymptomatic course for 4 years followed by a sensation of a sensitive palpable mass.

Known risk factors for RFO include emergency surgery, an unexpected change in the surgical procedure, and increased body mass index (8). While the first two are infrequent in aesthetic surgery, up to 36% of patients undergoing such operations have increased body mass index (>25) (9). The patient in our case had none of the mentioned risk factors: she had an elective surgery, with no unexpected changes in procedure, and her BMI was <25.

The surgical count is a fundamental element in surgical safety procedures and is encouraged by the World Health Organization (10). Accountable items include any reusable instrumentation or disposable items used during surgery, including surgical sponges, instruments, and sharps. Syringes and their caps, however, are often omitted from the count. The basic concept of “when in doubt – count” should always be applied.

## Conclusion

A retained foreign object is a rare iatrogenic complication, especially in breast augmentation procedures. A high level of vigilance should be maintained, even when complaints and imaging findings are non-specific. As pocket lavage is a common practice in breast augmentation and other surgeries, this report serves as a valuable reminder to alert surgeons to the hazard of retained foreign objects during operation. Surgical teams should either account for syringe covers and other disposable items at the end of operations or refrain from using them. A requirement to tag plastic syringes and covers with a radiopaque mark should be considered. Otherwise, plastic syringes should be accounted for in the end of operations and syringe covers should be banned from the operating room.

## Patient Perspective

The patient described her perspective 3 months post-operatively. Her main messages are summarized here, in the order with which they were expressed, as we think they are valuable for clinicians:

1. “The most annoying feeling was that no one believed me.”
2. “My doctors were reluctant to send me to imaging examinations, probably because they didn't believe that anything was wrong with me or maybe because of financial considerations.”
3. “It still surprises me that all the imaging examinations did not demonstrate the foreign body.”
4. “It was extremely important for me when finally, one of the doctors looked at the imaging tests' CDs and did not rely solely on the radiologist's printed interpretation.”
5. “It was exciting and touching when finally, someone believed me after 8 years.”
6. “The suffering was immense. I couldn't engage in sports and work out or even play Frisbee at the beach.”
7. “I feel much better and most of the times have no complaints. However, as strange as it is, and although I know that the foreign body is no longer there, I can sometimes still feel the pain, and even dreamt about the foreign body wondering in my breast.”
8. “This is a very traumatic event. I wonder if it wouldn't have been better to endure a worse problem or even an infection but to have been quickly diagnosed with a shorter torment time.”

## Data Availability Statement

The original contributions generated for the study are included in the article/supplementary material, further inquiries can be directed to the corresponding author.

## Ethics Statement

Written informed consent was obtained from the individual(s) for the publication of any potentially identifiable images or data included in this article.

## Author Contributions

CF, AM, and YW: design of the work. CF, AM, IW, SK, and YW: acquisition and analysis. CF, AM, IW, and YW: interpretation of data, have drafted the work, or substantively revised it. All author: have made substantial contributions to the conception, read and approved the manuscript, agreed to be personally accountable for their contributions and to ensure that questions related to the accuracy or integrity of any part of the work, even ones in which the author was not personally involved, are appropriately investigated, resolved, and the resolution documented in the literature.

## Conflict of Interest

The authors declare that the research was conducted in the absence of any commercial or financial relationships that could be construed as a potential conflict of interest.

## Publisher's Note

All claims expressed in this article are solely those of the authors and do not necessarily represent those of their affiliated organizations, or those of the publisher, the editors and the reviewers. Any product that may be evaluated in this article, or claim that may be made by its manufacturer, is not guaranteed or endorsed by the publisher.
